# The effects of introduced procedural errors on malaria rapid diagnostic test performance in a laboratory setting

**DOI:** 10.1186/s12936-025-05647-5

**Published:** 2025-12-24

**Authors:** Scott Wilson, Yong Ah, Michael Aidoo

**Affiliations:** 1 CDC Foundation, Atlanta, GA USA; 2 Laboratory Science and Diagnostics Branch, Division of Parasitic Diseases and Malaria, Centers for Disease Control and Prevention, Atlanta, GA USA

**Keywords:** Rapid diagnostic test, Sub-Saharan Africa, Community health worker, Instructions for use, Quality assurance

## Abstract

**Background:**

Rapid Diagnostic Tests (RDTs) are the primary means of malaria diagnosis in sub-Saharan Africa. Outside large health facilities, individuals performing RDTs have little or no formal laboratory training, and performing RDTs is by job aids derived from manufacturers’ instructions for use (IFU). Furthermore, in many countries, RDT products are interchanged often without associated training or notification to users of differences in characteristics not immediately obvious to non-laboratory workers. This leads to common deviations from IFUs the consequences of which have not been systematically studied. This study investigated how these errors impact RDT results.

**Methods:**

Six RDT products were tested using cultured *Plasmodium falciparum* diluted to represent infections with a range of parasitaemia. Tests were performed according to IFU (baseline) then with deviations from the IFU including changes in buffer volume, blood volume and incubation time. Effects of the deviations on test validity and overall test result compared to baseline were captured. Also captured, were effects of deviations on test band intensity, and ease of reading result due to test window abnormalities.

**Results:**

Increasing sample volume beyond the recommended 5µL impaired RDT performance, with 4 of 6 RDT products showing 83.3% (30/36) invalid results due to faulty sample migration. No invalid results were observed for the remaining two products. The shortest incubation period (5 min) led to the most deviations from baseline, whereas longer periods aligned more with baseline results. Insufficient buffer volumes caused at least one invalid outcome [10/27 (37%)] in over half the products. Conversely, exceeding buffer volumes led to reductions in the proportion of invalid results among all tests with 0 of 45 invalid results. Higher parasitaemia was associated with increased band intensity and resulted in the fewest deviations from baseline among all products. At 1,000 parasites/µL and 5 µL sample, three products achieved 100% agreement with baseline regardless of incubation time and buffer volume.

**Conclusion:**

Although malaria RDTs are tolerant of some errors, in general, procedural errors adversely affect results, particularly in low parasitaemia samples. Understanding how sample and buffer volumes, alongside incorrect incubation times, influence RDT performance can be incorporated into training and continuous quality improvement.

**Supplementary Information:**

The online version contains supplementary material available at 10.1186/s12936-025-05647-5.

## Background

Malaria continues to be a disease of significant global health concern, with an estimated 249 million cases and 608,000 fatalities recorded in 2022 across 85 endemic countries [1]. One pivotal advancement in the field of malaria diagnosis has been the development and widespread adoption of malaria Rapid Diagnostic Tests (RDTs). Malaria RDTs have revolutionized malaria diagnosis, offering a rapid, easily accessible, cost-effective means of diagnosis and disease management that also prevents the overuse of antimalarial drugs [2–4]. Traditionally, microscopic examination of blood smears has been the diagnostic standard, however, this approach has challenges in resource-limited settings [5, 6]. Malaria RDTs have quickly become a cornerstone in the global fight against malaria since their emergence in the 1990s [2, 3, 7–9].

Globally, 3.9 billion RDTs for malaria were sold by manufacturers between 2010 and 2022, with more than 82% of sales in sub-Sahara African (SSA) countries [1]. Malaria RDTs currently account for about 80% of all malaria diagnosis in SSA, reflecting the WHO recommendation that all suspected cases are confirmed using a parasitological test before treatment, and their relative ease of use [1, 10, 11].

The hallmark of RDTs lies in their accessible design, allowing most health workers, including those without formal medical laboratory training, such as community health workers (CHWs), to use them effectively. This simplicity has led to the widespread adoption of RDTs across diverse healthcare settings, from well-equipped tertiary hospitals to remote communities [12, 13]. However, the accuracy of RDT results depends on strict adherence to manufacturer instructions for use (IFU). Despite the importance of complying with manufacturer IFU, prior studies suggest that health workers throughout SSA receive limited training, leading to poor quality of testing [7, 14–16]. Challenges documented in studies include difficulties in accurately collecting and transferring the correct amount of blood using the kit supplied blood transfer device (BTD), applying incorrect number of drops of buffer, and prematurely interpreting test results [14, 17–23]. These IFU deviations are further complicated by the diversity of available RDT products, each characterized by a unique set of requirements, such as blood and buffer volumes used, blood collecting devices and incubation times [24–26]. The complexities associated with RDT use go beyond procedural adherence. Procurement practices, aimed at market stabilization, have led to frequent changes in RDT products in many countries, resulting in the simultaneous use of multiple products and rotation of products in a single country [24, 25, 27].

Minimal and inconsistent training, and the frequent introduction of different RDTs without proper notice or clarification of differences between products may contribute to poor RDT performance and potentially, misdiagnoses. This highlights the importance of quality control, adherence to IFUs, and ongoing training. Studies have shown that implementing training programmes and quality assurance systems ensures higher levels of performance and accuracy in RDT results [15, 16, 22, 23, 28, 29]. However, challenges persist in areas with limited training, underscoring the need for additional support especially in resource-limited health systems [30–36].

To better understand and strengthen the case for improved training and quality assurance systems, this study systematically investigated the impact of intentionally introduced procedural errors that deviate from manufacturer provided IFUs in a laboratory setting and how they influence RDT results. Operator errors identified in field settings, such as incorrect blood volume, incorrect buffer drops, and deviations from specified incubation times were tested. In addition, how these errors affect test accuracy based on sample parasite concentrations were examined.

## Methods

### RDT selection

Six RDT products that met the WHO malaria RDT procurement criteria [26] were selected. Selection was based on a Panel Detection Score (PDS) of ≥ 75% for *Plasmodium falciparum* samples at 200 parasites/µL, the performance threshold set by WHO for meeting procurement acceptability. The study exclusively focused on *P. falciparum* samples and, therefore, the PDS for non-*Plasmodium* parasites was not taken into consideration. All six RDT products were designed to detect histidine-rich protein-II (HRP2), with the additional test band identifying Pf-pLDH (n = 1), Pv-pLDH (n = 1), or PAN-pLDH (n = 4) antigens. Only HRP2 antigen bands were analysed in the study. The manufacturer recommended incubation times of the tests were 15 or 20 min, with variations in buffer volume between two and four drops. All products required a blood sample volume of 5µL. The same lot of each RDT product was utilized for both baseline testing and testing with introduced errors. A complete list of the RDTs and their characteristics is provided in Table 1.

**Table 1:**
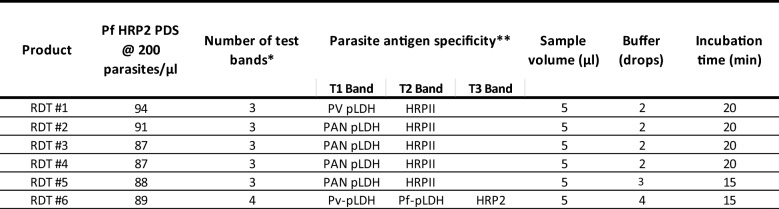
RDT products

List of the selected RDTs, and their PDS, target antigens and test parameters

^*^Number of bands including control bands

^**^Band arrangement begins with control moving from left to right

### Parasite sample preparation

*Plasmodium falciparum* strain US08FNigeriaXII sourced from the US Centers for Disease Control and Prevention (CDC) culture-adapted parasite repository was used throughout the study. The parasite was isolated from an imported malaria case to the US and previously adapted to in vitro culture by employing standard *P. falciparum* culture techniques [37]. Parasite antigen concentrations and parasite density were previously determined using enzyme-linked immunosorbent assay (ELISA) and 100X oil immersion Giemsa-stained smear microscopy, respectively. The antigen concentrations for the *P. falciparum* parasite preparation at 2,000 parasites/µL were 79.36 ng/mL for HRP2, 118.24 ng/mL for pLDH, and 11.63 ng/mL for aldolase. The prepared stock culture was diluted using a malaria parasite and antigen negative group O + human blood procured from a US blood bank (Interstate Blood Bank, Memphis, Tennessee, USA). The final parasite density preparations used for testing were 1,000 parasites/µL, 200 parasites/µL and 50 parasites/µL. Approximately 2 mL of each dilution was prepared in 50 µL aliquots distributed into 250 µL tubes. Aliquots were frozen at −80 °C until used. Each aliquot was thawed once for single use in one testing session and any leftover sample was discarded.

### Testing

RDT products were tested according to the manufacturer’s IFU to determine baseline result at each parasite density. A total of 240 conditions (3 parasite concentrations × 4 samples volumes, 5 buffer volumes × 4 read times) were tested per RDT product, including baseline. Recorded effects included those that changed the overall test result (e.g. a positive, negative, or invalid) and those that did not affect the overall result but affected the intensity of a positive test band were considered deviations.

Procedural errors were then introduced into subsequent testing for three parameters (sample volume, buffer volume, and incubation time). Table 2 outlines each of the conditions tested. Using a 20 uL micropipette, the initial sample volume tested was 5 µL (correct volume for all tests) then increased to 20 µL in 5 µL increments. The number of buffer drops were increased from 1 to 5 drops using separate tests for each increase in drops. Since the RDT products varied in the number of buffer drops required, the assessment of insufficient and excessive buffer drops is product specific. RDTs #1 through #4, which required two drops of buffer each exhibited one instance of insufficient volume (i.e. one buffer drop). By contrast, RDT #5, which required three drops, demonstrated two instances of insufficient buffer volume (i.e. one and two buffer drops), while RDT #6, requiring four drops, exhibited three instances of insufficient volume (i.e. one, two and three buffer drops). The result for each combination of buffer drops and incubation time was recorded in 5-min intervals using the same test—providing four recorded results for each test i.e., 5-, 10-, 15- and 20-min results.

**Table 2:**
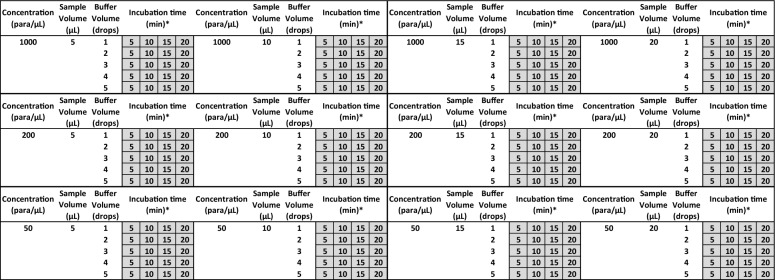
Testing conditions

List of all experimental combinations of sample volumes, buffer volumes, and read time intervals for each parasite density

^*^Each incubation time is an introduced error condition excluding the product's IFU baseline result (one baseline per conc.)

**Fig. 1:**
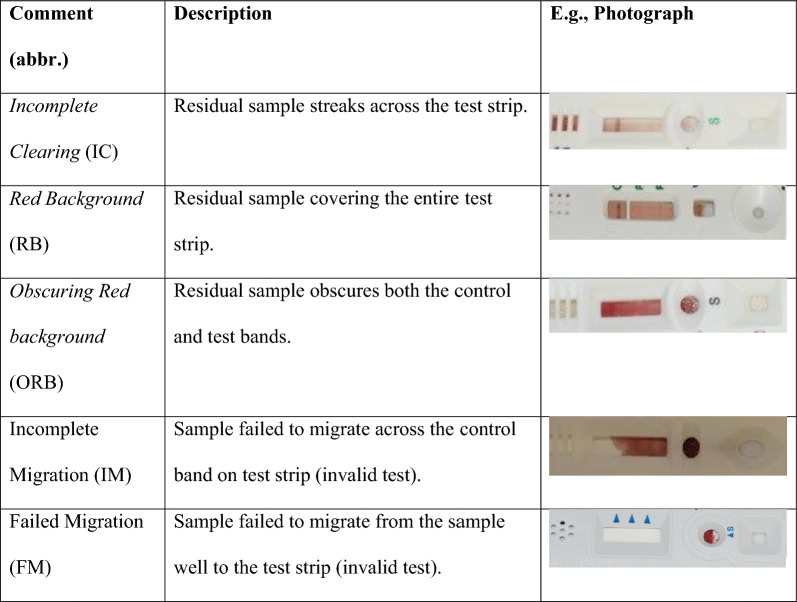
Observed anomalies. Depicts examples of anomalies and their descriptions encountered in this study

Results were recorded using the same system provided by WHO-Global Malaria Programme for the WHO product testing of malaria RDTs for both RDT reactivity and anomalies [26]. Test band intensity of a positive sample ranged from 0 to 4 using the same colour intensity template as in the WHO RDT evaluation, with 0 representing no reaction and 4 representing strongest reaction. Anomalies in the test window were identified and recorded during reading times. Examples of anomalies recorded are shown in Fig. 1 and include incomplete clearing (IC), red background (RB), obscuring red background (ORB), incomplete migration (IM), and failed migration (FM). In accordance with the IFU, any test without control band reactivity, regardless of the reactivity of the test line, was considered invalid. The results were entered into a heat map with colours utilized to distinguish whether a test outcome aligned with the baseline, deviated from it, gave a false negative, or was invalid. In the heat map, dark green represented baseline results, light green represented results that were the same as baseline for the HRP2 band, light orange represented results that were different from baseline, pink represented false negative results and red represented invalid tests.

## Results

Using the heat map, baseline results were compared with the outcomes under various introduced IFU deviations. Focus was only on the histidine-rich protein 2 (HRP2) testing band of each test, given the potential confounding effects of multiple bands in the RDTs used in this study. The following assessments were performed by comparing the results with introduced deviations to the baseline result (i.e., the IFU recommended parameters).

### Increasing sample volume (> 5 µL)

Initial examination of the heat maps (supplemental Fig. 1a–f) shows that when the sample volume is double or more than the recommended volume, the performance of RDTs is impaired, as illustrated by the red (invalid) highlighted sections of the heat map. Focusing on baseline parameters (i.e., buffer volume and read time), collectively, four out of the six products (RDTs #1, #2, #5, #6) yielded 83.3% (30/36) invalid outcomes for sample volumes ranging from 10 to 20 µL with 29 out of 30 (96.7%) of the invalid results caused by both incomplete migrations and failed migrations. Conversely, the combined results from the two remaining RDTs (RDTs #3 and #4) had zero invalid results with 72.2% (13/18) of tests matching baseline results. Notably, despite the low rate of invalid tests, all recorded results at the baseline buffer volume and incubation time for tests conducted with larger sample volumes were accompanied by anomalies (54/54), specifically by occurrence of incomplete migrations (IM), failed migrations (FM), incomplete clearings (IC) and red backgrounds (RB) (See comments column in supplemental Fig. 1a-f for observed anomalies).

Given the negative impact of higher sample volumes exceeding 5µL on the performance of these four RDTs (# 1, 2, 5, & 6), the following presented results focus on the effects at the baseline 5µL sample volume parameter.

### Incubation times

**Fig. 2:**
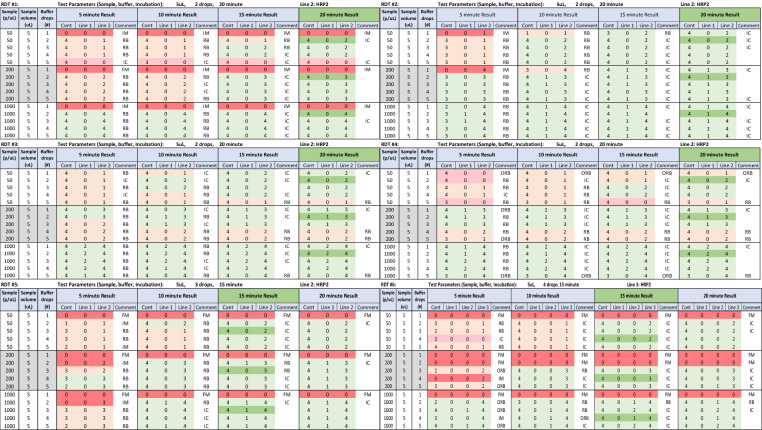
Sample Volume (5 µL) Results Heat Map. Heatmap showing a visual representation of RDT performance and behavior in response to user errors at 5 µL sample volume. As depicted for each product, incubation times are shown across the panel, while buffer drops, sample volume, and sample parasitemia (parasites/µL) are shown from top to bottom of each panel. In this scheme, traditional green signifies the baseline (testing according to manufacturer IFU) outcome, while light green signifies consistency with the baseline. Light orange indicates a deviation from the baseline, light red signifies a negative outcome for a positive sample, and traditional red denotes an invalid result including incomplete and failed migrations

Varying incubation times while testing with recommended buffer volumes revealed a trend where reading results earlier than recommended incubation times led to deviations from baseline results. While studying shorter incubation times, only two time-intervals (5- and 10-min) were observed for 15-min RDTs (#5 and #6) and three intervals (5-, 10-, and 15-min) were assessed for 20-min RDTs (#1–4). Considering all the products, early incubation times resulted in non-baseline results at a rate of 29.2% (14/48), with the majority of these being reduced band intensity. At the 5-min incubation time, one invalid and two negative results were observed. Additionally, anomalies were observed in nearly all tests at 97.9% (47/48) when read before reaching the baseline incubation period. These deviations are visualized in Fig. 2, where the orange and red tones are concentrated in the upper left-hand region of the heat map for each RDT product where results for shorter incubation times are shown. Conversely, baseline results for RDTs #1–4 at 5-, 10-, 15-min intervals were shown to increase with time at 58.3% (7/12), 75% (9/12), and 83.3% (10/12), respectively. Since RDT products #5 and #6 had a 15-min baseline incubation time, post-incubation effects were assessed on extending the read time by five additional minutes (20-min incubation period). Extending incubation time by five minutes resulted in tests all matching baseline results. Also, comparing the frequency of anomalies at the baseline 15-min incubation time with the additional 5-min read times reveals a decrease from 5 anomalies to 1, respectively.

Considering all the early incubation times, samples at 50 parasites/µL showed differences to baseline results at a higher rate of 62.5% (10/16) while the 200 and 1,000 parasites/µL samples showed non-baseline results at a rate of 18.8% (3/16) and 6.3% (1/16), respectively, reflecting the larger effect of errors on low density band intensities.

### Buffer volume

Product-specific variations in RDT performance were observed under conditions of insufficient buffer volumes. At insufficient buffer drops for RDTs #1, #5, and #6, 55.6% (10/18) of outcomes were invalid, while the remaining valid results were comparable to baseline. When subjected to only a single buffer drop, these three products had a 100% (9/9) invalid rate due to migration failures. In contrast, RDTs #2, #3, and #4 demonstrated better resilience to low buffer volumes with no invalids resulting and 88.9% (8/9) reaching baseline results.

By combining all the products’ specific testing parameters, a total of 27 instances of insufficient and 45 instances of excessive buffer volume occurrences were assessed. Compared with test results with insufficient buffer volumes, tests with excessive buffer volumes demonstrated no invalid test results [37% (10/27) vs 0% (0/45)]. Overall, excessive buffer volumes produced results consistent with baseline in 84.4% (38/45) of tests, with 13.3% (6/45) yielding non-baseline results and 2.2% (1/45) producing a false negative result. Additionally, the non-baseline results were exclusively derived from RDTs #3 and #4 at 33.3% (3/9) for both products. Furthermore, test window anomalies were observed in every occurrence (27/27) with insufficient buffer volumes. Anomalies were recorded for nearly half at 48.9% (22/45) in tests with excessive buffer volume.

### Parasite density

The impact of parasite density on RDT performance centered on changes to the band intensity of baseline results. As parasite density increased, there was a corresponding increase in intensity seen with the HRP2 testing band (Fig. 2).

Higher parasite density tended to mask adverse effects of introduced errors due to their association with higher band intensities. At 50 parasites/µL, the proportion of non-baseline results was higher at 49.1% (56/114), whereas it decreased to 25.4% (29/114) and 2.6% (3/114) at 200 and 1000 parasites/µL respectively. False negative test results were detected only at 50 parasites/µL, with an occurrence rate of 7.9% (9/114).

## Discussion

The main objective of the study was to determine the potential consequences on tests results when RDTs with different characteristics are often interchanged or concurrently used, especially by health workers, who lack formal laboratory training and may not realize subtle differences between RDT products. The results from this study demonstrate that while RDTs are relatively forgiving of certain errors, such as extended incubation times, other mistakes, like using too few drops of buffer or having shorter incubation times, either individually or in combination, may lead to higher rates of non-baseline test results. This study emphasizes the importance of adherence to product IFU and importance of end user knowledge of differences between tests especially in settings where products are often interchanged.

Results of this study indicate a clear negative impact on RDT performance when exceeding the recommended 5µL sample volume. In most products, the RDT either failed to migrate properly, rendering them invalid, or the excess sample volume generated an unclear test window background (recorded as anomalies) that interfered with interpreting results. These findings highlight the importance of collecting and transferring the correct amount of blood to generate accurate results. While this study utilized a micropipette for blood transfer, BTDs are typically provided in RDT kits and are designed to accurately collect a specific volume of blood sample from the patient when used as directed. These devices vary in design and performance characteristics, ranging from loops, pipettes, and capillary tubes to specialized inverted cups.

Certain BTD can be challenging to use and are susceptible to errors in sample volume measurement [15, 18, 19, 21, 38, 39]. A previous study found that the most challenging task for health workers was adding the blood sample using a BTD, with only 18 out of 63 workers performing this task correctly [17]. Even with training in the use of BTDs, health workers may still struggle to add the correct volume of blood. In practice, it falls upon these health workers to operate these devices properly to attain the required sample volume and ensure proper application on the test. Understanding the differences between various BTDs, training healthcare workers in their use, and knowledge of how increased sample volume impacts RDT performance are essential to performing RDTs effectively and ensuring reliable diagnostic test results.

This study showed a consistent trend where shorter incubation periods led to greater deviations from baseline results, while longer durations maintained baseline outcomes. Specifically, extending the incubation period beyond the minimum required time resulted in the two products matching baseline results, accompanied by an 80% reduction in anomalies. All time extensions for this study were still within the allowable read time specified by the manufacturer. Previous studies have highlighted that CHWs often tend to read test results too soon, despite reading the manufacturer’s instructions and job aids [15, 21, 38, 40]. Premature recording of test results was done as soon as the blood sample spread across the results window, immediately after the control band appeared, or even without following the proper timing guidelines. This practice may lead to inaccurate interpretations with higher variability in results [27]. RDT manufacturers specify a time window for minimum and maximum read times in their instructions for use (IFUs). Our findings suggest that allowing RDTs to fully incubate, and slightly extending the incubation period beyond the minimum recommended read time, while staying within the maximum limit, can improve accuracy, reduce anomalies, and enhance the reliability and interpretability of the results. This emphasizes the importance of adhering to incubation guidelines to optimize RDT performance.

The evaluation of RDT results with insufficient buffer volumes revealed product-specific variations, with certain products showing a 100% invalid rate due to migration failures. Understanding that RDT products react differently to insufficient buffer volumes emphasizes the need for careful attention to product-specific instructions. The number of buffer drops required can vary between RDTs, with some needing six drops while others only require two. Previous studies have demonstrated that CHWs, regardless of their training status or previous experience with RDTs, frequently apply an insufficient buffer volume despite manufacturer's guidance and job aids [7, 15, 16]. Conversely, results obtained with excessive buffer drops reduced the invalid rate compared to insufficient volumes.

While sample volume and buffer volume influence test results, their consequences differed. Increasing sample volume inhibited migration across the nitrocellulose strip rendering most tests invalid. By contrast, in some cases, increasing buffer volume slightly increased the number of tests that had similar test results as baseline results. When comparing all procedural components, at 5 µL sample volume, it is evident that lower parasite density samples were affected the most. Indeed, the only false negative results appeared at 50 parasites/µL in the early incubation times. These findings improve understanding of RDT operation and application of buffer/sample to tests. This could be useful in designing appropriate training, especially for non-clinical/laboratory RDT end users.

Although specific effects were product dependent, shorter incubation times, lower buffer volumes, and low parasite densities (translating to less discernible bands) tended to result in greater divergence from the baseline result, whereas reversing these factors tended to bring the results closer to the expected outcome. This trend is visually represented in the red and orange shading of the heat map, where the top left corner of panels for each RDT result represents lower values of incubation time, buffer volume, and parasite density, while the lower right corner signifies higher values of these parameters. Understanding the influence of parasite density in combination with other user errors is notable because, across all products, the highest deviations from baseline were recorded at 50 parasites/µL. Thus, lower density infections are more likely to yield a false-negative result. CHWs often misinterpret faint positive tests resulting from low parasitaemia as negative [41]. For example, a study mentions lighting conditions and poor eyesight can further complicate the ability to distinguish faint positive results from negative ones, even with proper training [15]. It is reasonable to conclude that low parasite density infections would be more prone to incorrect test results, especially in combination with errors demonstrated in this study. In general, RDT sensitivity is reduced at parasite densities < 200 parasites/µL and, therefore, operator errors when infections are characterized by low density parasitaemia coupled with other errors could compound the problem of producing inaccurate results.

The outcome of increased sample volumes causing migration failure for four of the six products caused much of the data to be impractical for analysis. The study could have been improved by incorporating lower incremental volume changes rather than increasing the sample volumes two-fold. This could have provided a more nuanced understanding of the sample volume threshold at which performance begins to deteriorate. Similarly, exploring the effects of decreased sample volume could have provided more understanding of its influence on test results, especially across low parasite density samples closer to the limit of detection of most RDTs. To further explore the observed trend of longer incubation times improving RDT results, more RDTs could have been read past their required incubation time. Another limitation of the study is that only six RDT products were investigated showing different product specific effects with the same procedural errors. Therefore, the consequences of errors described here are limited to the products used and suggest other products not tested in this study may behave differently under the same conditions. Nonetheless, the effects of errors described here provide information that can be used to emphasize adherence to manufacturer IFU and for additional considerations for training when products are being replaced within a country.

## Conclusion

Despite their simple and user-friendly design, this study revealed that RDTs can yield inaccurate and invalid results with deviations to test procedure, such as sample and buffer volume, and read times. It is important that operators perform the test properly as errors during key steps can adversely influence the quality of results directly affecting patient care and overall trust in RDTs. In addition, repeating many invalid tests due to user error could increase the operation cost of implementing malaria RDTs. Examining the operational characteristics in this study revealed a tendency for low parasite density samples to be disproportionately impacted especially with shorter incubation times. This study demonstrates the impact of user errors on RDT performance, providing valuable insights for developing new and/or improving training strategies, especially to accommodate countries that undergo frequent switching of RDT products. These findings emphasize the awareness needed when products are interchanged, and the importance of proficiency testing that incorporates elements of the different or newly acquired RDT characteristics and how they could influence results.

## Supplementary Information

Supplementary material 1.

## Data Availability

All experimental data are provided in the manuscript or supplemental material. Photos of all test windows are available upon request. RDT product identities are not provided as they were used off label based on the study design and irrelevant for the study objective.
