# Supplementary material for: The effects of introduced procedural errors on malaria rapid diagnostic test performance in a laboratory setting
Source: Malar J. 2025 Dec 24;24:447. doi: 10.1186/s12936-025-05647-5 (PMC12729159; doi:10.1186/s12936-025-05647-5)
Supplement: Supplementary file 1 — Supplementary material 1. [file 12936_2025_5647_MOESM1_ESM.docx]

**Supplemental Figure 1a: All Condition Results for RDT #1**

**Supplemental Figure 1b: All Condition Results for RDT #2**

**Supplemental Figure 1c: All Condition Results for RDT #3**

**Supplemental Figure 1d: All Condition Results for RDT #4**

**Supplemental Figure 1e: All Condition Results for RDT #5**

**Supplemental Figure 1f: All Condition Results for RDT #6**

*Supplemental Figure 1a-f is a heatmap showing a visual representation of RDT performance and behavior in response to user errors for all conditions. As depicted for each product, incubation times are shown across the panel, while buffer drops, sample volume, and sample parasitemia (parasites/µL) are shown from top to bottom of each panel.* *In this scheme, traditional green signifies the baseline outcome, while light green signifies consistency with the baseline. Light orange indicates a deviation from the baseline, light red signifies a negative outcome, and traditional red denotes an invalid result including incomplete and failed migrations.*
